# Signal averaging in cryogenic fast-field-cycling NMR experiments

**DOI:** 10.1038/s41598-026-50382-9

**Published:** 2026-05-12

**Authors:** Michael Jurkutat, Kajum Safiullin, Pooja Singh, Benno Meier

**Affiliations:** 1 Institute of Biological Interfaces 4, Karlsruhe Institute of Technology, Hermann-von-Helmholtz-Platz 1, 76344 Eggenstein-Leopoldshafen, Germany; 2 Institute of Physical Chemistry, Karlsruhe Institute of Technology, Fritz-Haber-Weg 2, 76131 Karlsruhe, Germany

**Keywords:** Biophysics, Physics

## Abstract

The spin-lattice relaxation time constant $T_1$ characterizes the equilibration of a spin system in a magnetic field with its environment, the lattice. Fast-field-cycling (FFC) relaxometry is dedicated to $T_1$ measurements at low field. $T_1$ frequently exhibits non-trivial field and temperature dependences, which give access to structural and dynamical information. Knowledge of $T_1(B, T)$ is also of central importance in nuclear spin hyperpolarization, where one often seeks to transfer spin polarized samples from the point of hyperpolarization to the point of detection. Here we present a new field control architecture for a unique FFC system which can probe relaxation properties between 0 and 2.5 T for temperatures from 300 down to 3 K. The field-profile is now defined directly by the NMR pulse sequence. PID control of the field improves measurement repeatability and reduces settling times, giving access to relaxation time constants down to 100 ms. The feedback control also decreases field errors to a degree that enables signal averaging and thereby the measurement of relaxation properties with improved sensitivity.

## Introduction

The subject of nuclear magnetic resonance (NMR) relaxometry is the dependence of the nuclear spin-lattice relaxation time constant $T_1$ on magnetic field and temperature^1^. Relaxation frequently is dominated by molecular motions that occur at or near the nuclear Larmor frequency. By varying the Larmor frequency, one can thus probe motion regimes that range from the fast motions of small moieties to the slow tumbling of large biomolecules. In addition to probing dynamics, varying the field often gives access to specific resonances that occur when a Non-Zeeman interaction is brought into tune with the Zeeman interaction. These resonances range from quadrupolar resonances in gels to the tunneling resonances of methyl groups^2–5^ and the recently reported non-secular resonances of dipolar spin systems^6^.

In many cases it is desirable to probe relaxation not only at different magnetic fields, but also at different temperatures. This applies in particular in the field of hyperpolarization, where nuclear spin hyperpolarization is often achieved at high field and temperatures as low as 1 K, but needs to be preserved efficiently at the higher temperatures and lower magnetic fields that the sample invariably encounters during transfer^7–11^. The transfer of such hyperpolarized samples can occur within less than 100 ms, and it is therefore desirable to probe relaxation times in that regime.

Since it is difficult to shuttle samples while maintaining temperature stability, the preferred way to investigate the field dependence of $T_1$ at low temperature is to rapidly vary the magnetic field itself. In the field-cycling apparatus described here, this is done via rapidly ramping the current in a superconducting coil.

Previously, this instrument suffered from poor repeatability, with field deviations in excess of 2 mT within consecutive cycles^6^, and substantial field variation even seconds after the setpoint had reached its final value. In addition, the instrument came with an obsolete Visual Basic control software that was cumbersome to operate and incompatible with modern NMR spectrometers.

Here we present a new control system that uses TTL signals to increase and decrease the field’s setpoint, thus allowing to program the time dependence of the field conveniently from within the NMR sequence using any NMR spectrometer with two TTL channels. We furthermore add a Hall sensor and a PID control loop, enabling a substantially faster and more accurate stabilization of the magnetic field, with variability reduced by an order of magnitude. Thus, the control architecture enables signal averaging for increased sensitivity^12,13^ and the detection of relaxation time constants down to 100 ms. This is demonstrated using 1-$^{13}$C-labeled pyruvic acid doped with 15 mM trityl radical OX063, a widely used marker molecule in DNP-applications ranging from NMR spectroscopy^14^ to *in vivo* human clinical imaging^15^.

## Results and discussion

### Limitations of the FFC apparatus

The field $B_0$ (0 to 2.7 T) in the superconducting coil is determined by the current (0 to 160 A) provided by the magnet power supply. The current can be set manually or set externally via a control voltage (0 to 10 V). In either case the precision of the actual field is limited ($\Delta B_0 = 2\,$mT) and field stabilization after the setpoint ramp is finished is slow ($\gtrsim 1$ s), see Fig. 2A and B. Both of these limitations can be alleviated substantially, as we show in this manuscript, by introducing a feedback control that adjusts the power supply’s external control voltage based on a measurement of the actual field from the superconducting coil.

### Feedback control system

**Figure 1:**
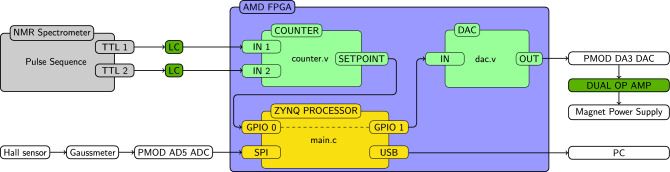
Simplified schematic of the control architecture. TTL channels on the NMR spectrometer are used to either increment or decrement the setpoint (TTL 1 and TTL2, respectively). Level converters (LC) are used to convert the 5 V logic of the NMR spectrometer to 3.3 V required for the FPGA. Inside the FPGA, a counter module written in Verilog increments or decrements the 16-bit setpoint depending on the TTL signals. The setpoint is passed to the ZYNQ processor via GPIO 0. When driving the system without feedback control, the microcontroller’s main.c simply sets GPIO 1 to match GPIO 0. Conversely, when applying feedback control, a Lakeshore HGCA-3020 cryogenic Hall sensor is used to determine the current field value employing a Lakeshore 475 DSP Gaussmeter. The Gaussmeter provides an analog output voltage that is proportional to the detected field. This voltage is digitized via a Digilent Peripheral Module (PMOD) AD5 using an Analog Devices AD7193 24-bit analogue to digital converter. The ADC is read out by the Zynq processor at a rate of approximately 1 kHz and averaged using an exponentially weighted moving average. A PID algorithm implemented in the Zynq processor is used to calculate a 16-bit control value that is provided at the GPIO 1 output port of the processor. In both operating modes, the GPIO 1 value is read by the dac.v Verilog module that controls the Digilent PMOD DA3, which is based on Analog Devices’ 16-bit AD5541A digital-to-analog converter. The output voltage of the DAC is corrected for offset and amplified three-fold using an AD823 dual-op-amp, and the resulting voltage is applied to the control input of the magnet power supply. The real time values of the ZYNQ processor’s inputs and its output are transferred to a PC via a serial port at a rate of approximately 10 Hz.

The control system is detailed in Fig. 1. The system is based on a Digilent Arty-Z7 20 prototyping board. This board hosts an AMD Zynq-7000 field-programmable gate array (FPGA). The FPGA comprises two domains. The Zynq processor domain is essentially a microcontroller that can be programmed in C in a similar way to the well-established Arduino platform. In addition, however, the FPGA programmable logic domain can support concurrent processes that can be programmed in a hardware description language (HDL), such as Verilog. A Verilog module is used to calculate the field setpoint based on two TTL signals that are provided by the NMR console. If and as long as TTL1 is low, it will increment the setpoint at a fixed rate, if TTL2 is low it will decrement the setpoint. The setpoint is a 16-bit integer that is provided to the Zynq processor via a general purpose input output port (GPIO 0). The actual field is measured via a Hall sensor and provided as an output voltage via the Lakeshore 475 DSP Gaussmeter. This voltage is digitized at a rate of approximately 1 kHz using an Analog Devices AD5541A 24-bit digital-to-analog converter (DAC), and the digitized voltage is filtered using an exponentially weighted moving average. The setpoint and the filtered voltage are used to calculate an error using a PID algorithm (the PID control code was written by Philip Salmony and is available for download at https://github.com/pms67/PID/) implemented in the Zynq processor. This error is used to adjust the Zynq processor’s output, which again is a 16-bit integer (GPIO 1). This output is wired to another Verilog module, which controls a 16-bit DAC with an output range of 0-2.5 V. Both the counter and the DAC module were packaged using a Makefile written originally by Pavel Demin (see https://doi.org/10.5281/zenodo.14543923 for the FPGA project that targets the Red Pitaya platform). The output of this DAC is corrected for offset and amplified to provide the 0-9 V output range needed to control the magnet power supply. The Zynq processor continuously reports its input and output values to a PC via a serial interface.

In order to characterize the systems performance without feedback control (corresponding to the configuration prior to the improvements described in this article), it is possible to bypass the PID loop by directly connecting the setpoint to the input of the DAC. For convenience this connection is implemented within the Zynq processor by setting the output GPIO 1 to equal the input GPIO 0. This connection is indicated by the dashed line in Fig. 1.

### Time evolution of the magnetic field without and with feedback control

**Figure 2:**
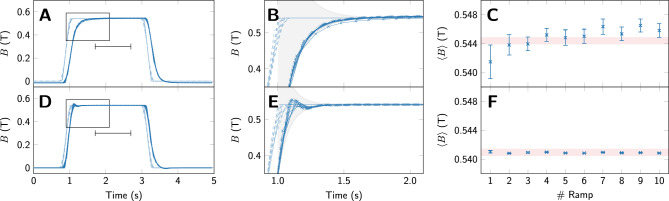
Time evolution of the magnetic field without (**A** - **C**) and with (**D** - **F**) feedback control. In panel (**A**) we show the setpoint (light blue, dashed) and the field as measured by the Hall sensor (dark blue) in 10 consecutive experiments. The chosen setpoint is the same in each experiment, and the apparent discrepancy between the setpoint curves is merely a sampling artefact. In panel (**B**) we show a zoom into the data from marked by the rectangle in panel (**A**). After the setpoint reaches the target field at 1 s, the asymptotic approach of the actual field is described by $\Delta B \sim 0.37\,\textrm{T}\cdot \text{ e}^{-(t-1\,\textrm{s})/0.13\,\textrm{s}}$ (shaded gray). It can be seen that substantial variability in the field between experiments persists long after the end of the ramp. This is also evidenced in panel (**C**), where we show, for each ramp the mean $\left\langle B\right\rangle$ of the magnetic field as measured in the interval 1.7 - 2.7 s with error bars representing one standard deviation. Conversely, when using PID feedback control, the deviation between field and setpoint is smaller and fades much faster (panel **E**), $\Delta B \sim 0.2\,\textrm{T}\cdot \text{ e}^{-(t-1\,\textrm{s})/0.08\,\textrm{s}}$. The repeatability is higher and the variability is approximately one order of magnitude lower (panel **F**), well within solid state NMR linewidths corresponding to $\approx$1 mT (shaded red).

Time profiles of 10 nominally identical field ramps without and with feedback control, are shown in Fig. 2 (A) to (C) and (D) to (F), respectively. In both cases, the setpoint was ramped from 0 to approximately 0.55 T, and the time evolution of the actual field was monitored with the Hall sensor. As can be seen in panel (A) and the magnified view in panel (B), without feedback control the field asymptotically approaches its final value. The setpoint ramp finishes at $t=1$ s, at which point the difference between setpoint and actual field is about 0.35 T. This difference decays exponentially with a time constant of about 0.13 s. As a rough measure of variability of the field in consecutive FFC NMR experiments, we plot in panel (C) the mean and standard deviation of the field determined for each ramp in the interval from 1.7 to 2.7 s. It can be seen that the mean varies by more than 2 mT, and the standard deviation varies from 2 to 4 mT reflecting a considerable drift due to the slow settling to a final value.

Conversely, when using feedback control (panels D - E) the field reaches its setpoint much faster and fluctuates much less between experiments. Here the difference between set and actual field is only 0.2 T when the setpoint settles at 1 s and the magnitude of this deviation decays much faster.

Note that the *y* axis range is the same in panels (C) and (F) for comparability. The standard deviations of the field are 2-3 mT without PID control, and 50 to 200 $\upmu$T with PID control, which should improve the performance in FFC-NMR experiments significantly. The measured field values for the measurements without feedback control (C) are larger overall than those for the measurements with feedback control (F). This discrepancy is due to the different meaning of the setpoint in these two modes of operation. Without feedback control, the setpoint defines the output voltage of the control circuit, which in turn regulates the current in the magnet. With feedback control, the setpoint defines the target value for the Hall sensor, and the output voltage is adjusted accordingly. We also indicate the typical NMR linewidth (shaded red) of about 1 mT, corresponding to 40 and 10 kHz for $^1$H and $^{13}$C, respectively.

### Field-cycling NMR experiments with feedback control

**Figure 3:**
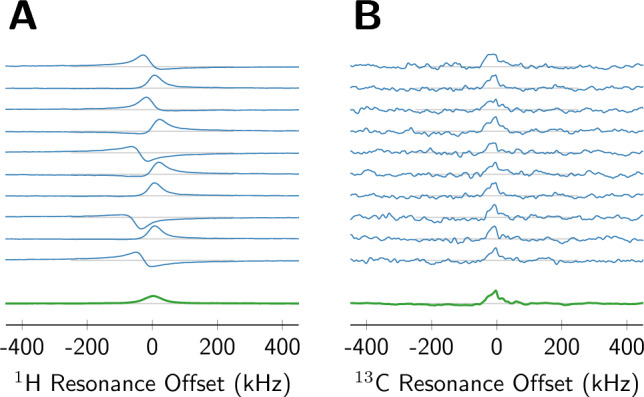
$^1$H (**A**) and $^{13}$C (**B**) NMR spectra of 1-$^{13}$C-pyruvic acid doped with 15 mM OX063 at 140 K after a field ramp with PID feedback control. All spectra were recorded at a carrier frequency of 23.42 MHz, corresponding to a detection field of 0.55 and 2.2 T for $^1$H and $^{13}$C, respectively. The top 10 spectra (blue) are randomly selected from a series of 100 consecutive recordings. The bottom spectrum (green) is the average of all 100 spectra. The $^1$H spectra exhibit substantial frequency offset deviations, and the SNR increases from 100 for an appropriately phased single spectrum to 320 for the average, i.e. approximately 3-fold. Due to the lower gyromagnetic ratio, the $^{13}$C spectra show a significantly smaller variability in phase and offset. The SNR increases from 5 to 32, i.e. approximately 6-fold.

Since a 200 $\upmu$T error corresponds approximately to a 10 kHz resonance offset for $^1$H, we went on to test the system’s suitability for NMR signal averaging. We conducted signal averaging experiments for both $^1$H and $^{13}$C on 1-$^{13}$C-labeled pyruvic acid doped with 15 mM trityl-OX063. In each experiment we recorded 100 scans, which may yield a gain in signal-to-noise ratio (SNR) up to $\sqrt{100} = 10$. Out of the 100 scans, we show 10 randomly selected spectra in Fig. 3, as well as the average of all 100 spectra. The same phase correction was applied to all spectra, since phase information cannot be extracted in cases where the single scan SNR is low. The SNR of the spectra was determined by dividing the maximum signal by two standard deviations of the noise, where the noise was measured in the frequency windows with $\pm \left[ 1, 2\right]$ MHz offset in the same way as described in Ref. ^13^.

For protons, the residual field error suffices to move the resonance by $\approx$ 20 kHz, such that the spectral range of the observed resonances is relevant compared to the excitation bandwidth of the 2 $\upmu$s $\pi$/2 pulse. Consequentially, the spectra exhibit varying phases^16^, and averaging them leads not only to a reduction of the noise by $\sqrt{10}$, but also to a visible reduction of the peak height. For protons, the gain in SNR is 3.2, significant, but well below the best-case value of 10.

Arguably, signal averaging is more important for low-$\gamma$ nuclei which are typically also less abundant than protons. In field-cycling experiments, the $^{13}$C signal is detected at an approximately 4 times higher field than the $^1$H signal. Conversely, the same absolute error in field leads to a four times smaller resonance shift for $^{13}$C compared to $^1$H. The $^{13}$C spectra in Fig. 3B show that signal averaging indeed works better. The individual spectra exhibit an SNR of 5, just above the limit of detection. The average spectrum, shown again in green, exhibits a peak height close to that of the individual spectra, with an SNR of 32, corresponding to an SNR gain of 6.4, reasonably close to the theoretical limit.

Encouraged by these results, we recorded $^{13}$C $T_1$ data, again using 1-$^{13}$C-labeled pyruvic acid doped with 15 mM trityl. The temperature was set to 40 K and we chose an evolution field of 0.05 T. For each trace, the sample was polarized for 60 s at 2.2 T where $T_1$ of $^{13}$C is 16 s. Then the field was ramped to the evolution field of 0.05 T for a duration of $t_\textrm{evo} =50$ ms, before it was ramped up to 2.03 T for detection. The signal was detected by applying a $\pi /2$ pulse 0.5 s after the setpoint had reached the detection field of 2.03 T. This experiment was repeated 32 times, and each trace was stored separately. In such a way sets of 32 traces were recorded for 12 different values of $t_\textrm{evo}$ in order to assess evolution of the longitudinal $^{13}$C magnetization for a broad range of delays.

**Figure 4:**
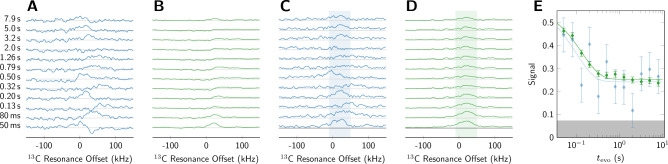
Comparison of $^{13}$C $T_1$ measurements in 1-$^{13}$C labeled pyruvic acid at 40 K and evolution field of 0.05 T, using 1 scan (blue) and 32 scans (green) per point. In panel (**A**), we show the real part of a single scan, with the evolution delay $t_\textrm{evo}$ indicated to the left of each trace. In panel (**B**) we show the real part of the average of all 32 acquisitions, indicating an NMR signal albeit with significant distortions. In panel (**C**) we show the magnitude of the spectra shown in (**A**), and in panel (**D**) we show the average of the magnitude of all 32 acquisitions. The data as shown in (**C**) and (**D**) are integrated (shaded area) to give the time-dependent intensities in panel (**E**). In the lowest panels of (**C**) and (**D**), as well as in (**E**) gray boxes mark the baseline of the background magnitude. The signal intensity in panel (**E**) is normalized to the signal from equilibrium polarization at 1 T. Without relaxation during the ramps or settling times one would observe the signal deacaying from equilbrium at polarization field to that of evolution field, i.e. from 2.2 to 0.05. Due to fast relaxation during the ramps, however, signal contrast is significantly diminished to relaxation from 0.45 to 0.25.

The resulting traces are shown in Fig. 4. In panel (A) we show the real part of a single spectrum recorded for each delay. In panel (B) we show the average of all 32 spectra recorded for each delay. While these spectra show an NMR signal, the phase is distorted, presumably due to the need to detect the signal shortly after the setpoint has reached its final value. Since the magnitude is insensitive to such phase variations, we plot in panel (C) the magnitude of the single spectrum recorded for each delay, and in panel (D) the average of all 32 magnitude spectra. The magnitude averages in (D) show a clear decay of the signal. In order to estimate $T_1$, we integrate the magnitude spectra in the indicated region (-10 to 50 kHz). We characterize the baseline (indicated by the gray boxes) by calculating the mean and standard deviations of the baseline integrals across the interval (-500 to -250 kHz).

In panel (E) we plot the signal intensity from the integrals of the magnitude spectra in the shaded region region for the single scan example and the averaged spectra. The errorbars indicate ±2 standard deviations of the respective baselines. The solid curves show fits according to the model

1 $$\begin{aligned} S(t_\textrm{evo}) = A\exp (- t_\textrm{evo}/T_1) + B, \end{aligned}$$

resulting in estimate of $T_1$ of $0.10\pm 0.09$ s for the single scan example and $0.13\pm 0.02$ s for the 32-scan average.

This is a clear demonstration that the implementation of the feedback control now allows for the measurements of longitudinal magnetization relaxation times down to 100 ms which is crucial for the studies of the DNP samples at low magnetic fields. The limiting factor for the measurement of fast relaxation at low fields is loss of contrast due to spin-relaxation during the ramps down to and back up from evolution field. This is evidenced in Fig. 4E where without relaxation during the ramps a 2 T polarization would decay to 50 mT. However, due to relaxation during the ramps we observe signal decaying from the field equivalent of 0.45 to 0.25 T. We note that the Hall sensor employed in this work is not a very accurate probe of magnetic field strength. The Hall voltage shows a significant temperature-dependence, and is - by NMR standards - not a linear function of the actual field, necessitating a future calibration of the Hall sensor by NMR at various field- and temperature points. From this point of view, an excellent standard for measuring field-strengths under cryogenic conditions is $^3$He^17,18^, which also exists as the endofullerene $^3$He@C$_{60}$^19,20^. However, the use of $^3$He has been discarded at this point, since we do require real-time field-information across a large field-range, that is translated into control output with minimum latency.

## Conclusion

The system reported here is able to rapidly stabilize the field to the desired value within 200 $\upmu$T, enabling signal averaging for low-$\gamma$ nuclei with substantial gains in signal-to-noise ratio. For $^{13}$C, the SNR gain from averaging 100 scans is $6 \approx 100^{0.4}$, instead of the theoretical limit of $100^{0.5} = 10$. While in this work we used pyruvic acid with a 14 M concentration of $^{13}$C, the increase in SNR should enable us to study relaxation in typical DNP samples with 1-3 molar concentrations of labeled glassing agents as they are frequently used in dissolution-DNP^14,21^.

We also demonstrate the ability to measure nuclear magnetization relaxation times $T_1$ as short as 100 ms using the field feedback control scheme. These improvements widen the scope of research capabilities decisively, and have enabled investigations of faster processes with higher resolution. Ongoing efforts in our laboratory target rapid relaxation in nitroxide-based DNP sample formulations and applications of the non-secular resonances we reported recently^6,22^.

A further improvement of the feedback loop through the Hall sensor calibration may increase the performance of the FFC apparatus. One may employ DNP to increase the starting polarization well beyond its thermal equilibrium value at 2 T, increasing the contrast and thereby extending the measurable range towards even shorter relaxation times.

## Materials and methods

### Hall sensor

A Lakeshore HGCA-3020 cryogenic InAs and GaAs Hall sensor was used to determine the magnetic field value. The operating temperature range of 1.5-375 K and magnetic field range of 15 T allows to use it during the FFC experiments. The nominal sensitivity of the sensor is 0.0669 mV/T. The sensor was located on the magnet’s symmetry axis 40 mm away from the center of the magnet and calibrated by means of NMR measurements. The sensor was used in combination with a Lakeshore 475 DSP Gaussmeter.

### Field-cycling NMR

The fast-field-cycling setup has been described previously^23^. It allows to perform NMR measurements at temperatures between 3 and 300 K. The magnetic field in the field-cycling experiments may be varied between 0 and 2.5 T and can be ramped at a rate of up to 10 T/s. 1-$^{13}$C-labeled pyruvic acid doped with 15 mM trityl radical OX063 was used as a test sample. In the reported experiments the NMR probe was tuned to 23.42 MHz and the magnetic field was adjusted in order to record NMR signals from $^1$H or $^{13}$C. In the $^{13}$C $T_1$ experiments, detection was carried out at 21.7 MHz or 2.03 T. The field was first ramped to 2.03 T, where the carbon signal was saturated. Then the field was ramped to 2.2 T (for increased polarization), and the polarization was built up over 60 s. Subsequently the field was ramped to 50 mT, where it was kept for the variable evolution delay before ramping back to 2.03 T for read out. A 0.5 s delay at high field was chosen to stabilize the field prior to read out. The experiment was repeated 32 times for each evolution delay. We note that the Hall sensor readout is not perfectly linear, such that the required ramp duration has to be determined prior to the experiment. Using the $^1$H calibrated ramp rate, we had to ramp to a nominal field of 2.063 T to get an actual field of 2.026 (corresponding to the $^{13}$C resonance frequency of 21.7 MHz).

## Data Availability

The NMR raw data are available at the KITopen repository at https://doi.org/10.35097/uk4sb464fq4uv23m. The Verilog module for control of the digital-to-analog converter is available at https://github.com/bennomeier/PmodDA3/.
